# Supplementary material for: Assembly of a unique membrane complex in type VI secretion systems of Bacteroidota
Source: Nat Commun. 2024 Jan 10;15:429. doi: 10.1038/s41467-023-44426-1 (PMC10781749; doi:10.1038/s41467-023-44426-1)
Supplement: Supplementary file 1 — Supplementary Information [file 41467_2023_44426_MOESM1_ESM.pdf]

a

Protein name	Prottertopology	TM (number. limits)
TssN	N(out)-C(in)	5 (14-152)
TssQ	N(in)-C(out)	1 (12-36)
TssO	N(in)-C(out)	1 (20-42)
TssP	N(in)-C(out)	1 (9-27)
TssR	Lipoprotein (OM)	SPII (1-22)

b

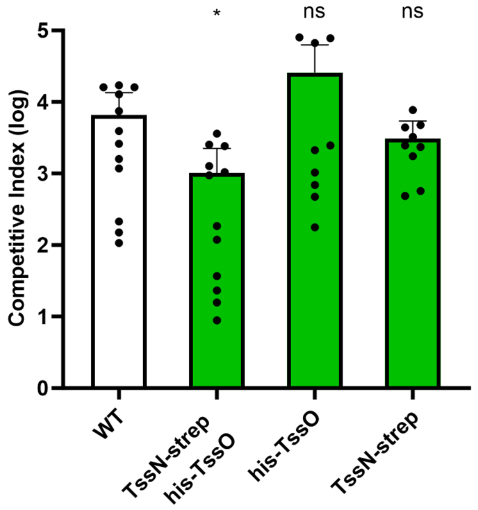

c

Protein	Dali hits
TssN	PDB Hit : 7b9f Z-Score : 9.7 RMSD : 2.6 Aligned residues : 87/288 (184-287) %ID : 13<br>Description : EccD Mycobacterium T7SS
TssQ	PDB Hit : 6wc3 Z-Score : 8.7 RMSD : 2.5 Aligned residues : 81/167 (65-151) %ID : 6<br>Description : Protein transport TIP20
TssO	PDB Hit : 7zn7 Z-Score : 9.4 RMSD : 2.8 Aligned residues : 83/175 (16-146) %ID : 4<br>Description : DNA Damage-binding protein
TssP	PDB Hit : 2y72 Z-Score : 11.7 RMSD : 1.4 Aligned residues : 75 (32-117) %ID : 27<br>Description : PKD Domain - Surface layer protein - Collagenase
TssR	PDB Hit : 5bv8 Z-Score : 14.1 RMSD : 3.7 Aligned residues : 177 (302-546) %ID : 10<br>Description : Von Willerbrand Factor

d

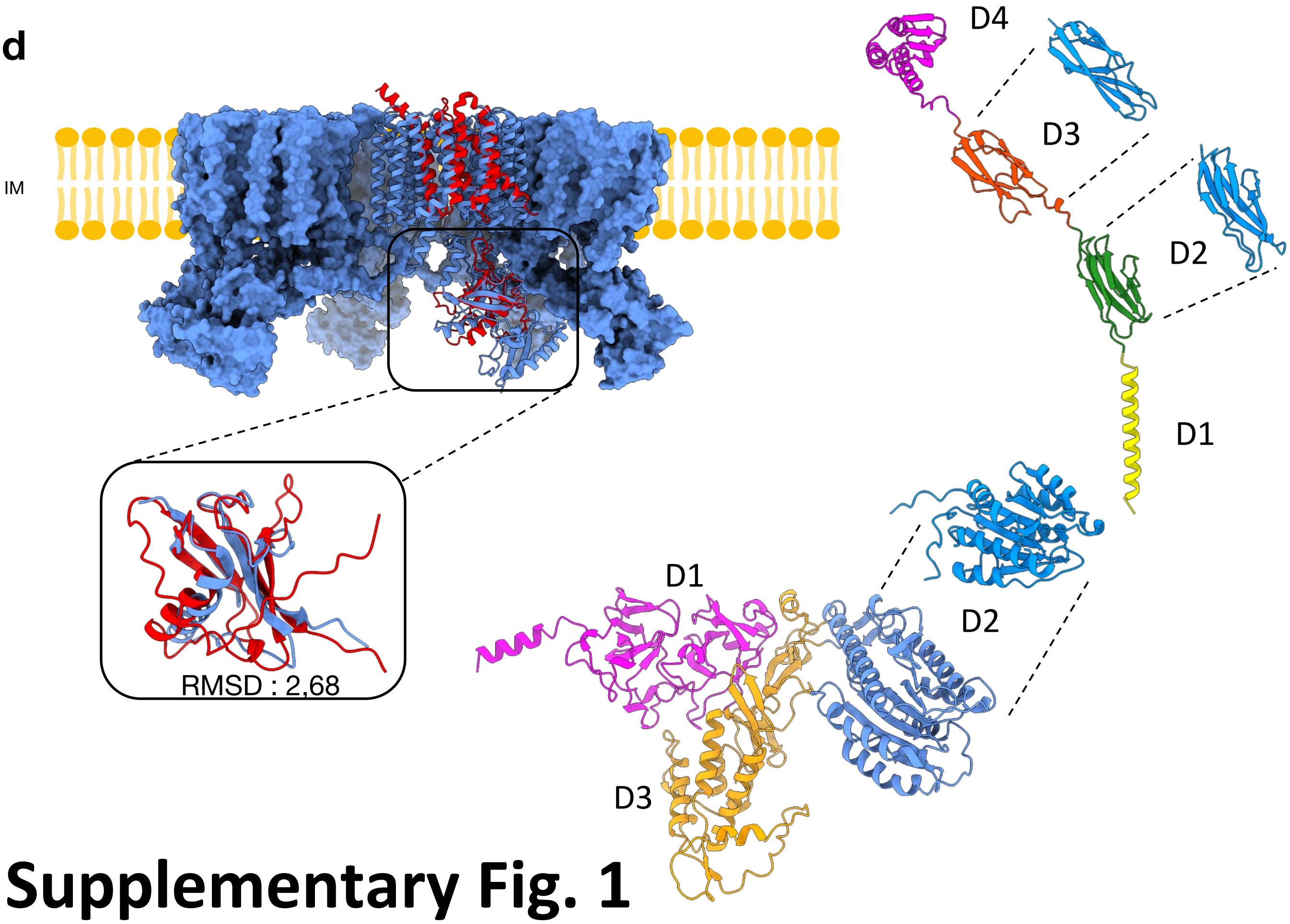

Supplementary Fig. 1

Supplementary Figure 1. Predicted topology and homology of the membrane complex. a, Table summarizing the results of the Protter webserver prediction and SignalP webserver prediction showing the membrane topology of TssN, TssQ, TssO, TssP and TssR. **b,** Competitions of *B. fragilis* wildtype, TssN-strep his-TssO, TssN-strep, and his-TssO versus *B. thetaiotaomicron*. In-frame chromosomal deletions of *tssN* and/or *tssO* were complemented in-frame with tagged TssN and/or TssO. Competitive index calculated as the ratio of (donor *B. fragilis*/recipient *B. thetaiotaomicron)*$_{\text{final}}$ / (donor *B. fragilis*/recipient *B. thetaiotaomicron*)$_{\text{initial}}$ CFUs. Mean ± s.d. are shown; graphs show ≥ 3 independent replicates, dots show means of individual biological replicates each with 3 technical replicates. * indicates P value = 0.01, ns indicates non-statistically significant, unpaired t tests. **c,** Dali webserver output showing the structural alignement of the TssNQOPR models to their best hits from the PDB. **d,** Illustration of the structural alignement of TssN with its homolog, EccD from the T7SS, TssP D2 and D3 with PKD domain and TssR D2 with the Von Willerbrand Factor.

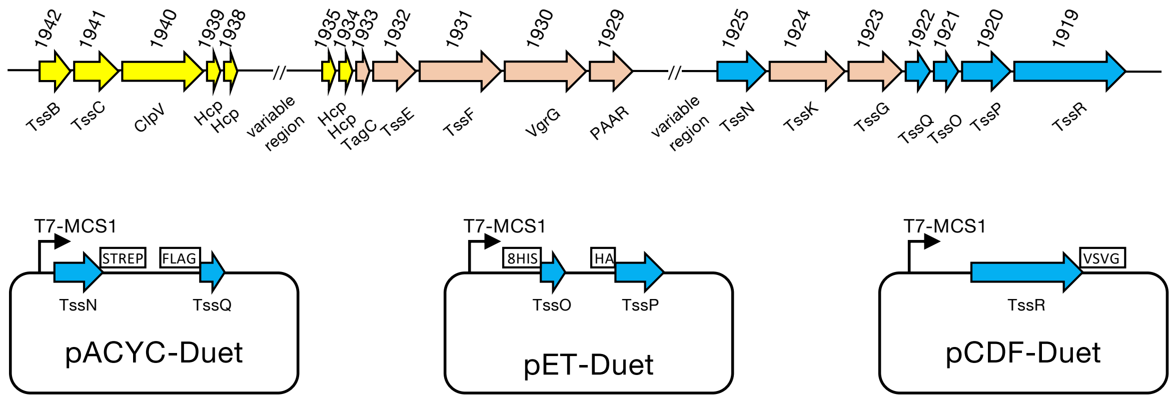

Supplementary Fig. 2

Supplementary Figure 2: Plasmid combination used to produce the TssNQOPR complex in E. coli BL21 (DE3). A combination of 3 plasmids were used, pACYC TssN^{STREP} ^{FLAG}TssQ, pET ^{H}TssO ^{HA}TssP and pCDF TssR^{VSVG}.

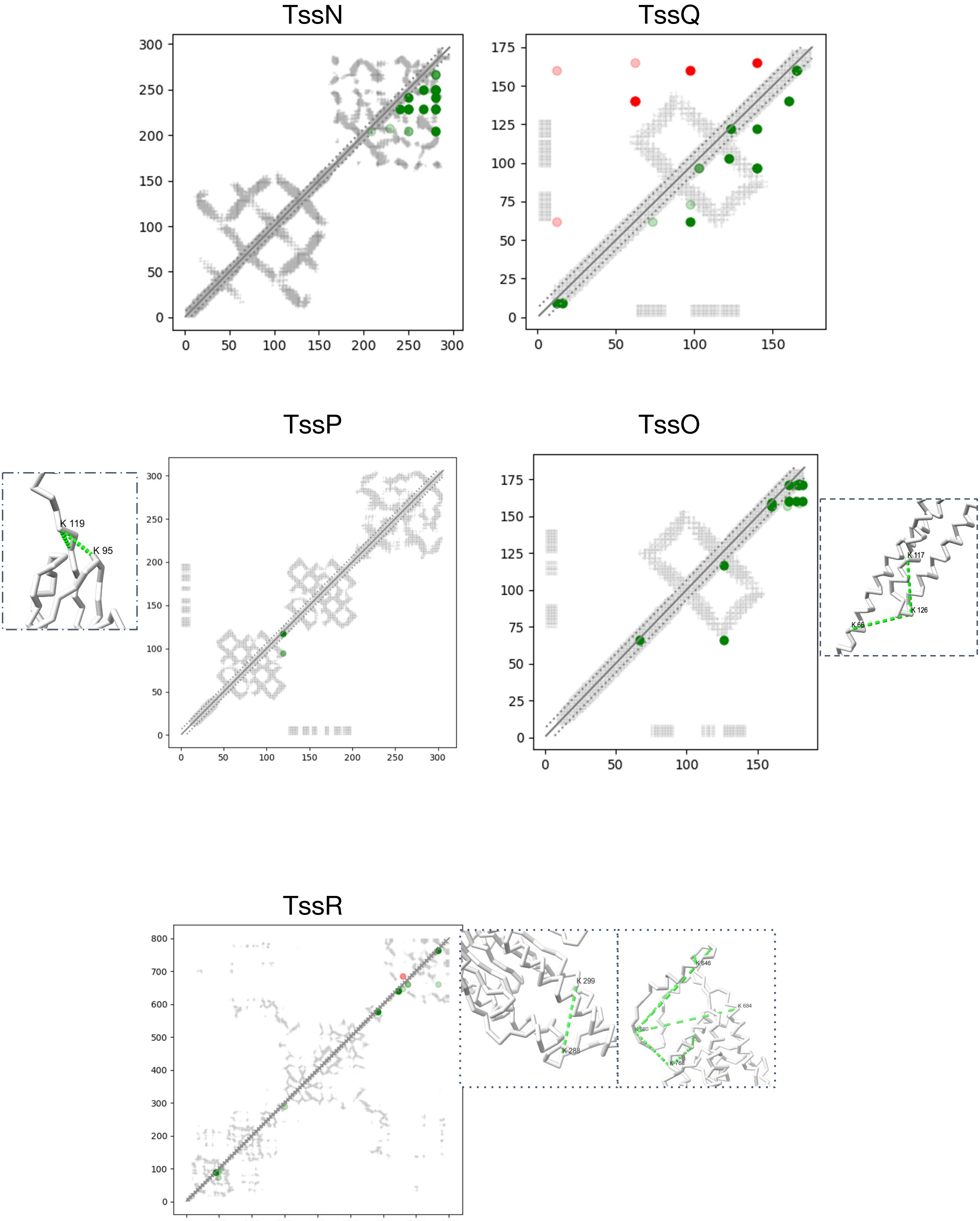

Supplementary Fig. 3

Supplementary Figure 3. Validation of predicted models using XL-MS.

Contact maps of predicted structures (grey dots) are superimposed with crosslinked residue pairs (green or red circles). x and y-axis are the residue indexes of the T6SS membrane complex sequence, and a grey dot is present if the distance between two residues is below 10 Ang. Structurally satisfied crosslinks between two residues (ie, the distance is below 35 Ang) and structurally violated crosslinks (above 35 Ang) are represented by green and red circles, respectively. High confidence (ie, ld score > 35) and low confidence (ie, 25 < ld score < 35) crosslinks are represented by solid and transparent colors, respectively. Insets depict the local structure of crosslinks satisfied between distant residue indexes.

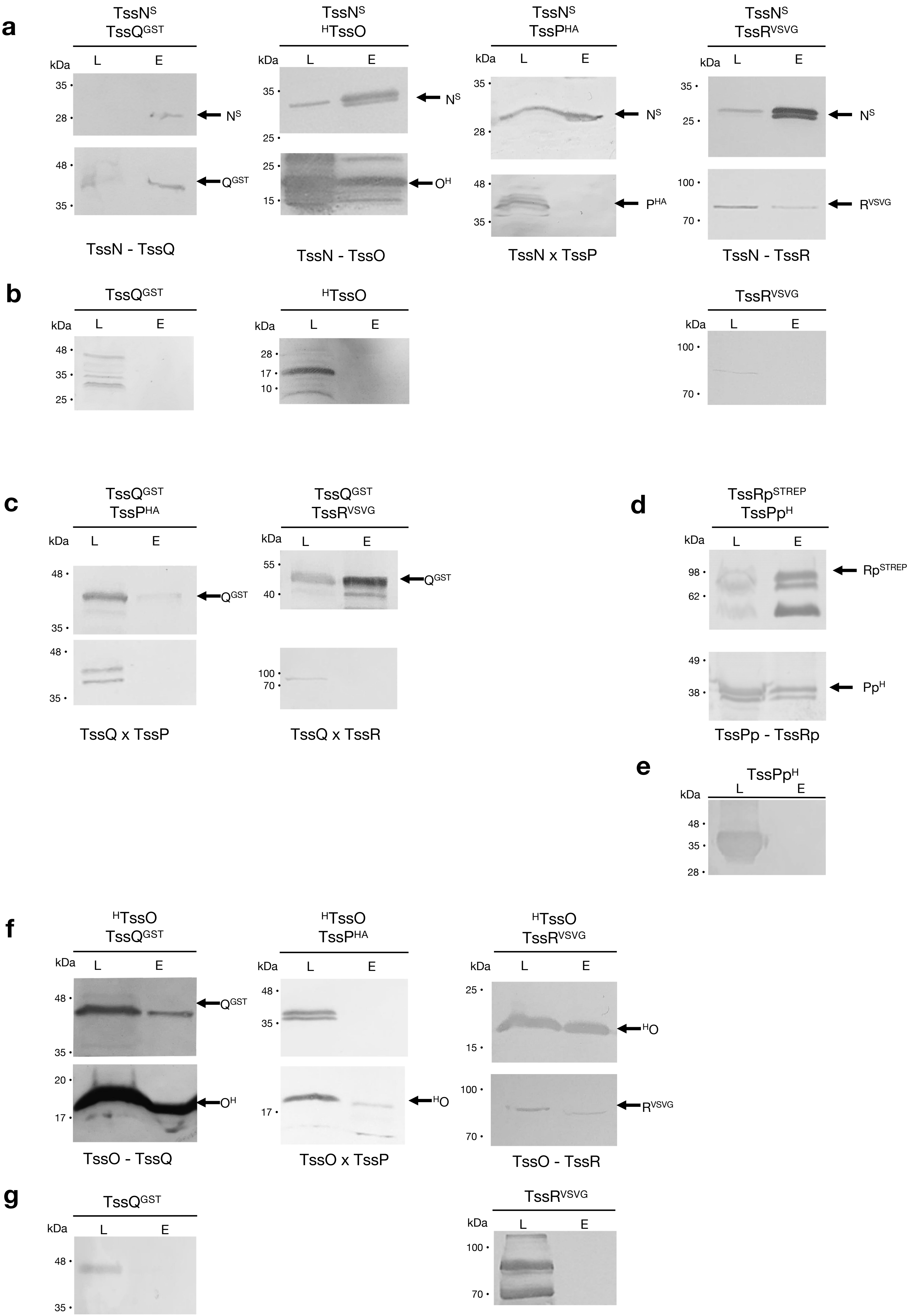

Supplementary Fig. 4

Supplementary Figure 4. Protein-protein interaction network determination. a, TssN interaction network. TssN copurifies with TssQ, TssO, and TssR, but does not copurify with TssP. **b,** Negative controls of the A panel copurifications, showing no purification of TssQ, TssO, and TssR on their own. **c,** TssQ interaction network showing no direct interaction with TssP and TssR. **d,** TssR and TssP soluble fractions copurification showing an interaction. **e,** TssPp by itself isn't purified on the TssRp-TssPp copurification conditions. **f,** TssO interaction network showing that TssO interacts with TssQ and TssR but not with TssP. **g,** Negative controls of copurification showing no purification of TssQ and TssR on a HISTRAP column.

a

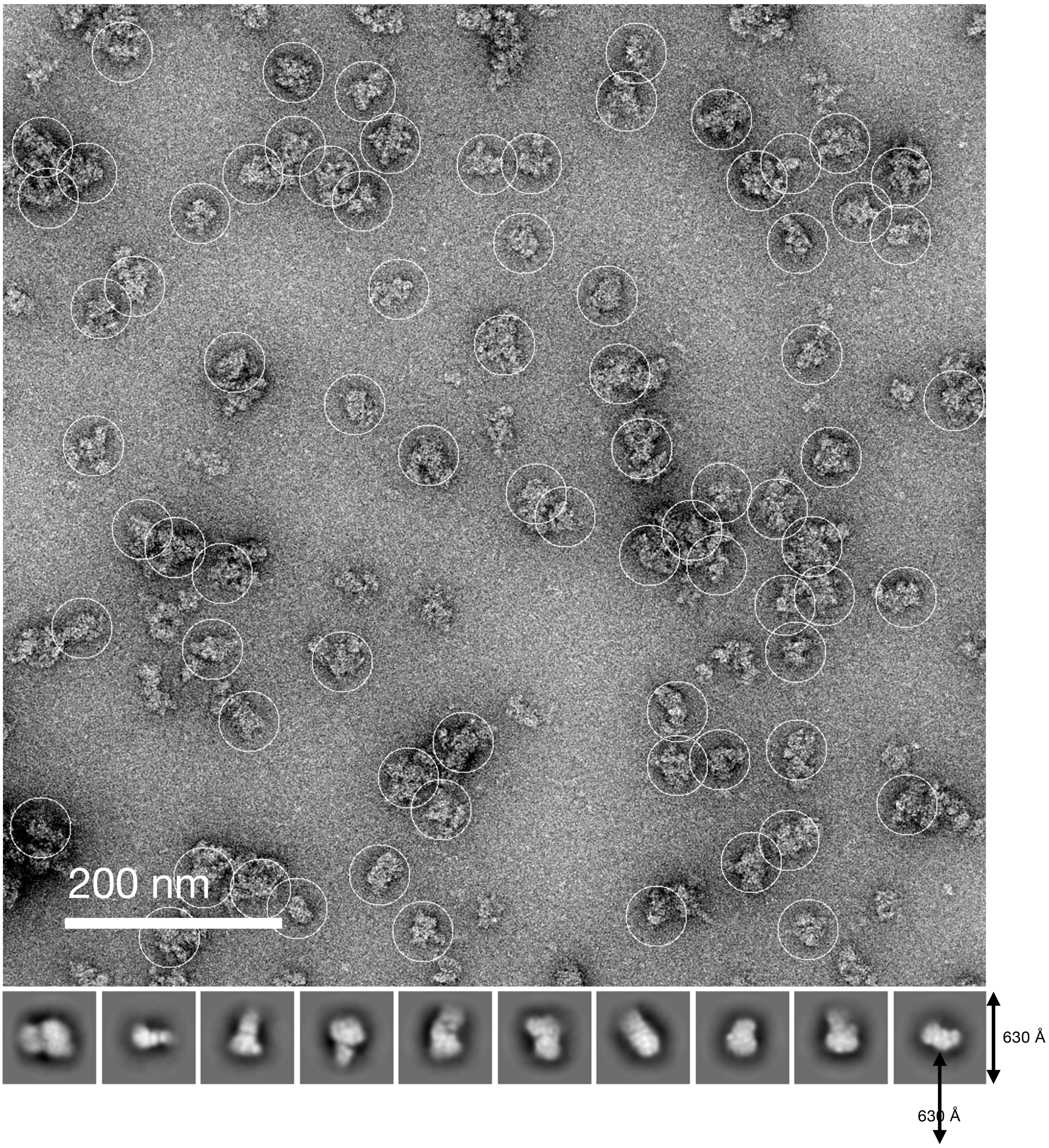

b

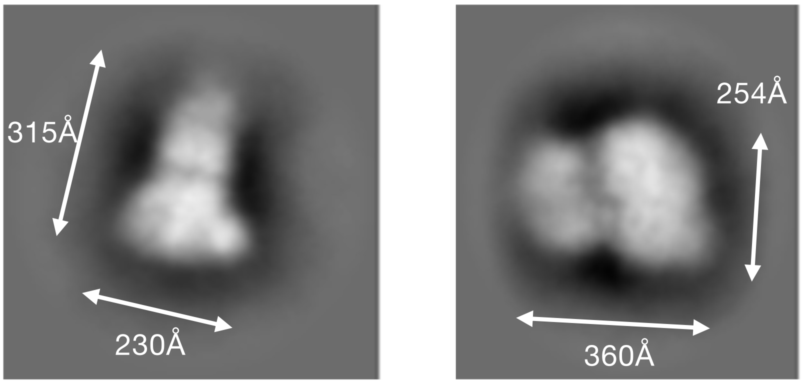

Supplementary Fig. 5

Supplementary Figure 5: TssNQOPR micrograph and membrane complex size determination. **a,** Picked particles and 2D classes from a negative staining grid of the TssNQOPR complex purified from *E. coli* overproduction. **b,** Particles size estimation obtained from 2D classification particles.

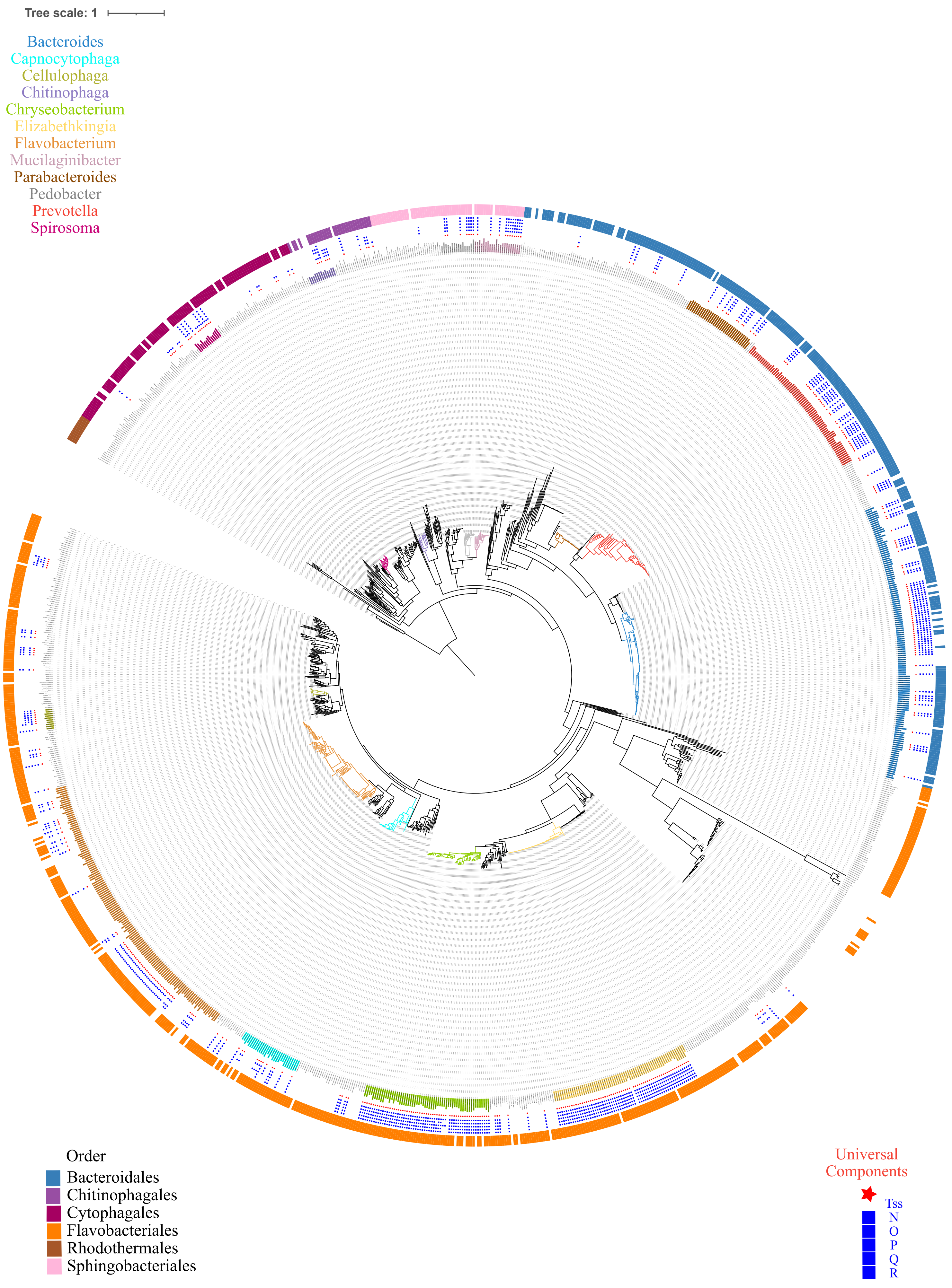

Supplementary Fig. 6

Supplementary Figure 6. **Presence of T6SS in the tree of all the genomes in the phylum.** The outer layers indicate the presence or absence of: T6SS (universal markers in just one group) and each of the MC components (see inset legend). The outer ribbon indicates the Order. The most represented genera are coloured according to the colors indicated in the inset legend.

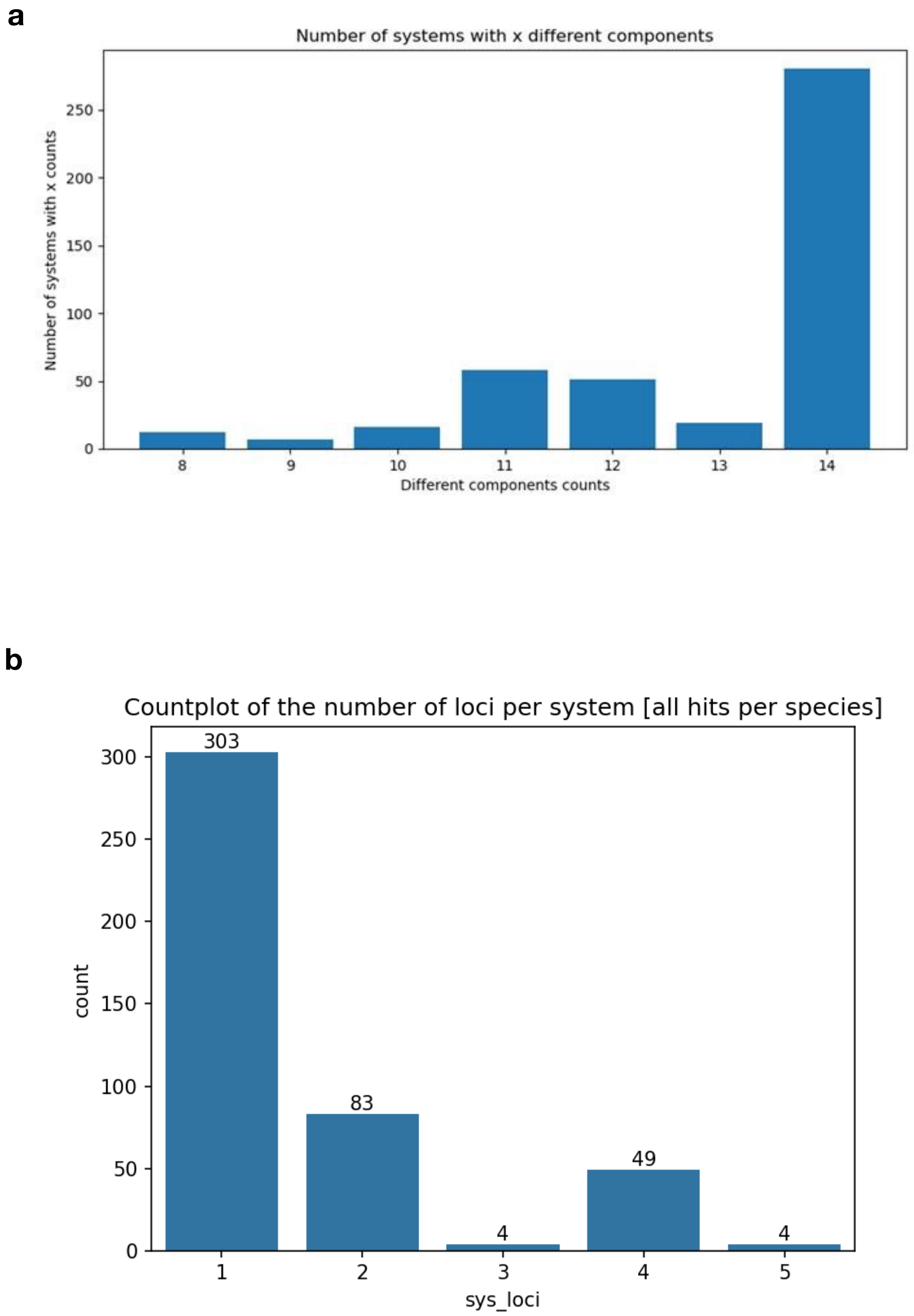

Supplementary Fig. 7

Supplementary Figure 7. a, Histogram of the number of different components per T6SS[iii] (out of 14 defined components in the MacSyFinder complete model). **b,** Histogram of the number of loci encoding a given T6SS.

Protein	Total(a)	Satisfied gap>20 (b)	Satisfied (c)		Violated (d)	
			HC (e)	MC (f)	HC (e)	MC (f)
TssN	13	8	9	4	0	0
TssO	15	2	12	3	0	0
TssQ	18	3	9	3	3	3
TssP	2	1	1	1	0	0
TssR	16	2	5	10	2	0

Supplementary Table 1. Validation of predicted models using XL-MS. (a) total number of identified and unique crosslinks. (b) number of satisfied crosslinks between residues with a sequence distance (gap) >20. (c) Satisfied crosslinks, ie, the distance between the C-alpha atoms of the residues is lower than 35 Ang. (d) Violated crosslinks, ie, the distance between the residues is higher than 35 Ang. (e) high-confidence crosslinks, ie, the ld-score is higher than 35. (f) medium/high-confidence crosslinks, ie, the ld-score is lower than 35 and higher than 25.

Supplementary Table 2. Strains, plasmids and oligonucleotides used in this study.

Strains

Strains	Description and genotype	Source
E. coli S17-1	thi pro hdsR hdsM+ recA, chromosomal insertion of RP4-2(Tc::Mu Km::Tn7), AmpS	PMID: 6340113
E. coli PIR-1	F-, Δlac169 rpoS(Am) robA1 creC510 hsdR514 endA recA1 uidA(ΔMluI)::pir-116	ThermoFisher
DH5α	F-, Δ(argF-lac)U169, phoA, supE44, Δ(lacZ)M15, relA, endA, thi, hsdR	Laboratory collection
BL21 (DE3)	*fhuA2 [lon] ompT gal (λ DE3) [dcm] ΔhsdS λ DE3 = λ sBamHIoΔEcoRI-B int::(lacI::PlacUV5::T7 gene1) i21 Δnin5*	*New England Biolabs*
B. fragilis NCTC 9343	*Δtdk*	ATCC25285, gift from Goodman lab, Yale. PMID: 26957597
B. fragilis NCTC 9343	*Δtdk* att1::pNBU2-ermGb	This study
B. thetaiotaomicron VPI-5482	*Δtdk* att1::pNBU2-tetQ-BC01	This study
B. fragilis NCTC 9343	*Δtdk* TssN-Strep His-TssO (chrom fusion nomenclature)	This study
B. fragilis NCTC 9343	*Δtdk BF9343_1941 (tssC)* att1::pNBU2-ErmGb	This study
B. fragilis NCTC 9343	*Δtdk BF9343_1925 (tssN)* tssN att1::pNBU2-ErmGb	This study
B. fragilis NCTC 9343	*Δtdk BF9343_1922 (tssQ)*	This study
B. fragilis NCTC 9343	*Δtdk BF9343_1921 (tssO)*	This study
B. fragilis NCTC 9343	*Δtdk BF9343_1920 (tssP)*	This study
B. fragilis NCTC 9343	*Δtdk BF9343_1919 (tssR)*	This study
B. fragilis NCTC 9343	*Δtdk BF9343_1925 (tssN)* att1::pNBU2-ErmGb_BT1311-TssN	This study
B. fragilis NCTC 9343	*Δtdk BF9343_1922 (tssQ)* att1::pNBU2-ErmGb_BT1311-TssQ	This study
B. fragilis NCTC 9343	*Δtdk BF9343_1921 (tssO)* att1::pNBU2-ErmGb_BT1311-TssO	This study
B. fragilis NCTC 9343	*Δtdk BF9343_1920 (tssP)* att1::pNBU2-ErmGb_BT1311-TssP	This study
B. fragilis NCTC 9343	*Δtdk BF9343_1919 (tssR)* att1::pNBU2-ErmGb_BT1311-TssR	This study
B. fragilis NCTC 9343	*Δtdk* att1::pNBU2_ErmGb_BT1311-TssNc	This study
B. fragilis NCTC 9343	*Δtdk* att1::pNBU2_Erm_P1T$_{DP}^{A21}$-TssNc	This study
B. fragilis NCTC 9343	*Δtdk BF9343_1925 (tssN)* att1::pNBU2_ErmGb_BT1311-TssNc	This study
B. fragilis NCTC 9343	*Δtdk BF9343_1925 (tssN)* att1::pNBU2_Erm_P1T$_{DP}^{A21}$-TssNc	This study
B. fragilis NCTC 9343	Δtdk ΔBF9343_1942 (tssB) att1::pNBU2_Erm_P1T_DP-A21-TssB-sfGFP	This study
B. fragilis NCTC 9343	Δtdk ΔBF9343_1942 (tssB) ΔBF9343_1924 (tssK) att1::pNBU2_Erm_P1T_DP-A21-TssB-sfGFP	This study
B. fragilis NCTC 9343	Δtdk ΔBF9343_1942 (tssB) ΔBF9343_1925 (tssN) att1::pNBU2_Erm_P1T_DP-A21-TssB-sfGFP	This study

B. fragilis NCTC 9343	Δtdk ΔBF9343_1942 (tssB) ΔBF9343_1922 (tssQ) att1::pNBU2_Erm_P1T_DP-A21-TssB-sfGFP	This study
B. fragilis NCTC 9343	Δtdk ΔBF9343_1942 (tssB) ΔBF9343_1921 (tssO) att1::pNBU2_Erm_P1T_DP-A21-TssB-sfGFP	This study
B. fragilis NCTC 9343	Δtdk ΔBF9343_1942 (tssB) ΔBF9343_1920 (tssP) att1::pNBU2_Erm_P1T_DP-A21-TssB-sfGFP	This study
B. fragilis NCTC 9343	Δtdk ΔBF9343_1942 (tssB) ΔBF9343_1919 (tssR) att1::pNBU2_Erm_P1T_DP-A21-TssB-sfGFP	This study

Plasmids

Vectors	Description	Source
Vectors for manipulating *B. fragilis*		
pNBU2-tetQ-BC01	Tetracycline resistance in *B. thetaiotaomicron* competition	PMID: 24439897
pNBU2-ermGb	Erythromycin resistance in *B. fragilis* competitions	PMID: 18611383
pNBU2-ErmGb_BT1311	Erythromycin resistance and constitutive overexpression promoter	PMID: 24439897
pNBU2_Erm_P1T_DP-A21	Erythromycin resistance and constitutive overexpression promoter	PMID: 28431252
pNBU2-ErmGb_BT1311-TssN	Constitutive moderate expression of TssN for complementation assays	This study
pExchange-Δtdk-ΔTssC	Deletion of BF9343_1941 (tssC)	This study
pExchange-Δtdk-ΔTssK	Deletion of BF9343_1924 (tssK)	This study
pExchange-Δtdk-ΔTssN	Deletion of BF9343_1925 (tssN)	This study
pExchange-Δtdk-ΔTssQ	Deletion of BF9343_1922 (tssQ)	This study
pExchange-Δtdk-ΔTssO	Deletion of BF9343_1921 (tssO)	This study
pExchange-Δtdk-ΔTssP	Deletion of BF9343_1920 (tssP)	This study
pExchange-Δtdk-ΔTssR	Deletion of BF9343_1919 (tssR)	This study
pExchange-Δtdk-TssN-Strep	Deletion of BF9343_1925 (tssN) and chromosomal reintegration of TssN-Strep	This study
pExchange-Δtdk-His-TssO	Deletion of BF9343_1921 (tssO) and chromosomal reintegration of His-TssO	This study
pNBU2_erm_P1T_DP-A21-TssB-sfGFP	Constitutive expression of TssB-sfGFP	This study
Vectors for protein overproduction		
pACYC-Duet1	Expression vector, lacI, PT7, CmR	Addgene
pET-Duet1	Expression vector, lacI, PT7, AmpR	Addgene
pCDF-Duet1	Expression vector, lacI, PT7, SMR	Addgene
pRSF-Duet1	Expression vector, lacI, PT7, KanR	Addgene
pACYC-TssN^{S}	TssN-StrepII cloned into pACYC-Duet1	This study
pACYC-TssN^{S}-Q^{F}	TssN-StrepII, FLAG-TssQ cloned into pACYC-Duet1	This study
pACYC-$^{S\text{-}S\text{-}TEV}$TssNc	StrepII-StrepII-TEV-TssNc cloned into pACYC-Duet1	This study
pET-TssO^{H}	TssO-8xHIS cloned into pET-Duet1	This study
pET-TssO^{H}-TssP^{HA}	TssO-8xHIS, HA-TssP, cloned into pET-Duet1	This study
pET-TssP^{HA}	HA-TssP cloned into pET-Duet1	This study

pCDF-TssK^{H}	TssK-8xHIS cloned into pCDF-Duet1	This study
pCDF-TssK^{HA}	TssK-8-HA cloned into pCDF-Duet1	This study
pCDF-TssR^{VSVG}	TssR-VSVG cloned into pCDF-Duet1	This study
pRSF-TssN^{HA}-^{F}TssQ	TssN-HA, FLAG-TssQ cloned into pRSF-Duet1	This study
pRSF-^{GST}TssQ	GST-TssQ cloned into pRSF-Duet1	This study

Oligonucleotides

Name	Destination	Sequence (5'→3')

For biochemistry experiments (pCDF/pRSF/pET/pACYC)

pACYC-TssN^{S} Insertion of the *Bacteroides fragilis* (BF9343_1925) TssN sequence into pACYC-Duet1, C-terminal Strep-II epitope
FW: ATTCCATGGAAGGAGATATACATATGaattcattcttccaaaccctcgccttcacctacctgctctacccg
REV: TATGGATCCTTATTTTTCGAACTGCGGGTGGCTCCAgttctcattatgttccattacccgtttagc

pACYC-TssN^{S}-^{F}Q Insertion of the *B. fragilis* (BF9343_1922) TssQ sequence into pACYC-TssN^{S}, C-terminal FLAG epitope
FW: ATAGGATCCAAGGAGATATACATatgGATTATAAAGATGACGATGACAAGaaagcaaagaacagtgagaaaatcatccgtgg
REV: AATGAGCTCTTActtttctacactaatgtttcccaatgtcagctttcgggtagcctgtttattgtcctgaatgc

pACYC-$^{S\text{-}S\text{-}TEV}$TssNc Insertion of the *B. fragilis* (BF9343_1925 162 to 288) TssNc sequence into pACYC-Duet1, N-terminal Strep-II Strep-II Tev
FW1: CCACCCGCAGTTCGAAAAAGAGAACCTCTACTTCCAATCGccgattcccgtgtatgaag
FW2: TGTTTAACTTTAATAAGGAGATATACCATGGgaTGGAGCCACCCGCAGTTCGAAAAATGGAGCCACCCGCAGTTCGA
REV: CATTATGCGGCCGCAAGCTTttagttctcattatgttccattacccg

pET-TssO^{H} Insertion of the *B. fragilis* (BF9343_1921) TssO sequence into pET-duet1, C-terminal 6xHIS epitope
FW: ATTGAGCTCAAGGAGATATACATatg CATCACCATCATCACCACCATCACagcataacccctaaaaataaaagcagcaaaaccg
REV: ATTCTGCAGtcatggcttgcctcctttatttttaagttcgccttccttcttctggagtccgacttcgcactcc

pET-TssO^{H}-TssP^{HA} Insertion of the *B. fragilis* (BF9343_1920) TssP sequence into pET-TssO^{H}, N-terminal HA epitope
FW: ATACTGCAGAAGGAGATATACATatgTACCCATACGATGTTCCAGATTACGCTatgaataaattcctaaatacgaagg
REV: AATGTCGACtcatttcttttttcgatttgcataaccgttatttgggtaatatatccgctctcttcatcc

pET-TssP^{HA} Insertion of the *B. fragilis* (BF9343_1920) TssP sequence into pET-duet1, N-terminal HA epitope
FW: ATACTGCAGAAGGAGATATACATatgTACCCATACGATGTTCCAGATTACGCTatgaataaattcctaaatacgaagg
REV: AATGTCGACtcatttcttttttcgatttgcataaccgttatttgggtaatatatccgctctcttcatcc

TssN-Strep Cloning Strep tag into TssN locus (BF9343_1925)
FW: AACATTCGAGTCGACGAATTCATTCTTCCAAACCCTCG
REV: TTTCGAACTGCGGGTGGCTCCAGTTCTCATTATGTTCCATTACCC

pCDF-TssR^{VSVG} Insertion of the *B. fragilis* (BF9343_1919) TssR sequence into pCDF-Duet1, C-terminal VSV-G epitope
FW: ATTGTCGACAAGGAGATATACATatgagaaagttatttgtcctattgatttctatcgtcgttttatcggc
REV: TATGCGGCCGCctaTTTTCCTAATCTATTCATTTCAATATCTGTATAcggtaacagttctgaagctatgtagtaatagc

pRSF-TssQ-GST Insertion of the *B. fragilis* (BF9343_1922) TssQ sequence into pRSF-Duet, C-terminal GST epitope
FW: ATACCATGGAAGGAGATATACATatgtcccctatactaggttattg
RV: taaGAGCTCttacttttctacactaatgtttcc

For gene chromosomal deletions

pExchange-ΔtdK-ΔTssN Used for the deletion of the *B. fragilis* BF9343_1925 gene
FW1 : ATATCTAGAAACTTTGGAGGTATAGTTATTTTAATTTCCGAGACAAATGGG
FW2 : TCGAAATATGAATTCAGAGAACTAAGAATATAAAAATAAAACTACATACTATATGGC
REV1 : GTTAATTTAAAAACAAAAGTCGAAATATGAATTCAGAGAACTAAGAATATA
REV2 : CGCGGCGAACTGGGTGTGCACATCTCTCTGATATGTCGGGTCGACATT

pExchange-Δtdk-ΔTssQ Used for the deletion of the *B. fragilis* BF9343_1922 gene
FW1 : ATAGGATCCGGCATACATAGAACTAAAGACCAACCGCCGGGGG
FW2: AAGAATTATGAAAGCAGAAAAGTAAAATAGGAAAGATATGAGCATAACCCC
REV1: GGATAAACACTTATTTATAAGAATTATGAAAGCAGAAAAGTAAAATAGGA
REV2 : GCGAGAATCTGGTACTGGGAGTTCGGAAACAACGGTCGACATT

pExchange-Δtdk-ΔTssO Used for the deletion of the *B. fragilis* BF9343_1921 gene
FW1 : ATAGGATCCTGAGGCTGATTTGCATATACACATAGGCGACCTGCC
FW2 : GAAAGATATGAGCATAATAAAGGAGGCAAGCCATGATGAATAAATTCC
REV1 : GTGTAGAAAAGTAAAATAGGAAAGATATGAGCATAATAAAGGAGGCAAGCC
REV2 : CGAACACGCTGGTTATTGTCAACAACAATAAATATAACGGTCGACATT

pExchange-Δtdk-ΔTssP Used for the deletion of the *B. fragilis* BF9343_1920 gene
FW1 : ATAGGATCCACTTATCGAGGAACTGCAAATAATGGATAGC
FW2 : GCAAGCCATGATGAATATGCAAATCGAAAAAAAGAAATGAAATGAGAAAG
REV1 : GGCGAACTTAAAAATAAAGGAGGCAAGCCATGATGAATATGCAAATCGAAAAAA
REV2 : GCGTTTTCCAGAGTGCCGGCTTTGCAAAAGACGCTGAAGTCGACATT

pExchange-Δtdk-ΔTssR Used for the deletion of the *B. fragilis* BF9343_1919 gene
FW1 : ATAGGATCCGGGAAGAAATATTTTTTCCGATTCCACGTCGG
FW2 : AAATGAAATGAGAAAGTTACCGTAGGAGAAAAAGTATTTTCCAAAGAAAAATAGCG
REV 1: CGGTTATGCAAATCGAAAAAAAGAAATGAAATGAGAAAGTTACCGTAGGAGAAAA
REV 2: CCTATTTTCTTTGCTTGGTACTATTATTTCACATCCTGGTTCAATGTCGACATA

pExchange-Δtdk-ΔTssK Used for the deletion of the *B. fragilis* BF9343_1924 gene
FW1 :gaaagaagataacattcgagtcgacctatactattggtgcaatcac
FW2 : tatggcaaagatggattgatatggaacatcgc
REV1 : atcaatccatctttgccatatagtatgtagttttatttttatattc
REV2: cggccgctctagaactagtggatccgcaaatcagcctcagcac

For overexpression experiments (pNBU2)

pNBU2_ErmGB_BT1311 Used for the overexpression of TssNc
FW: CTCCAAATCTGTTTTTAACAATGCCGATTCCCGTGTATG
REV: GCCCGGGGGATCCACTAGTTTTAGTTCTCATTATGTTCCATTACCC

pNBU2_Erm_P1T$_{DP}^{A21}$ Used for the overexpression of TssNc
FW: TTTATGATATTAAACGAATCATGCCGATTCCCGTGTATG
REV: CTGGAAGATAGGCAATTAGTTAGTTCTCATTATGTTCCATTAC

For fluorescence experiments (pNBU2)

pNBU2_erm_P1T_DP-A21-TssB
FW : tttatgatattaaacgaatcATGGCAATACTTGAATATGG
REV: cgcctttacgtccacctcctgcagctgcTTCGGTCTTTAATTCGTTAC

pNBU2_erm_P1T_DP-A21-sfGFP
FW: aaagaccgaagcagctgcaggaggtggaCGTAAAGGCGAAGAGCTG
REV : actggaagataggcaattagTCATTTGTACAGTTCATCCATAC

Supplementary Table 2. Strains, plasmids and oligonucleotides used in this study.
