# Supplementary material for: Assembly of a unique membrane complex in type VI secretion systems of Bacteroidota
Source: Nat Commun. 2024 Jan 10;15:429. doi: 10.1038/s41467-023-44426-1 (PMC10781749; doi:10.1038/s41467-023-44426-1)
Supplement: Supplementary file 4 — Source data files [file 41467_2023_44426_MOESM4_ESM.zip › Source Data Files/Uncropped_WB.pdf]

Native pulldown

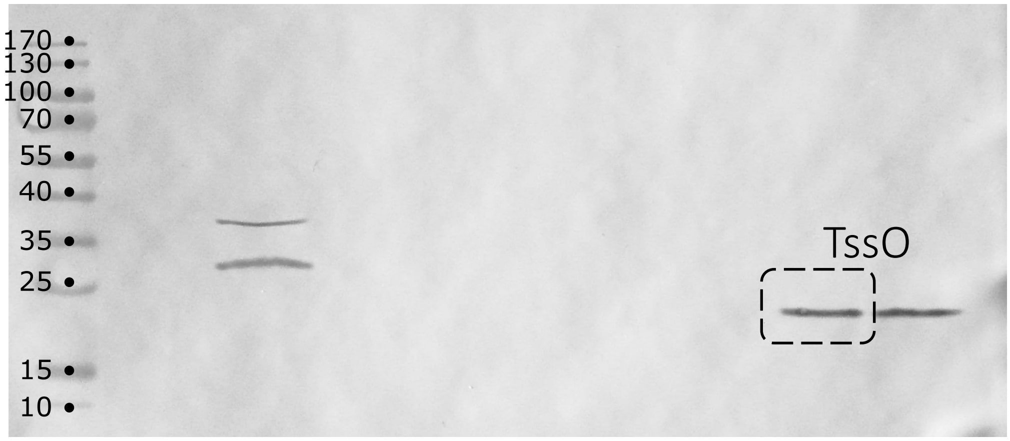

α-HIS

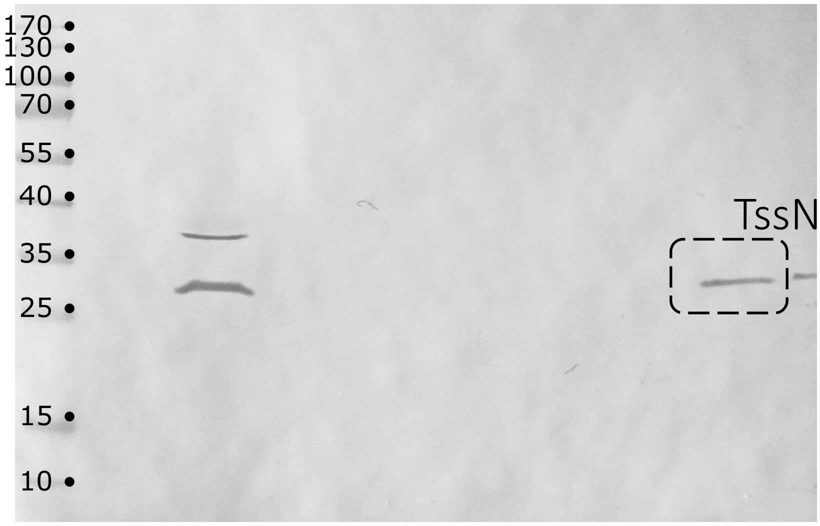

α-STREP

Figure 2b

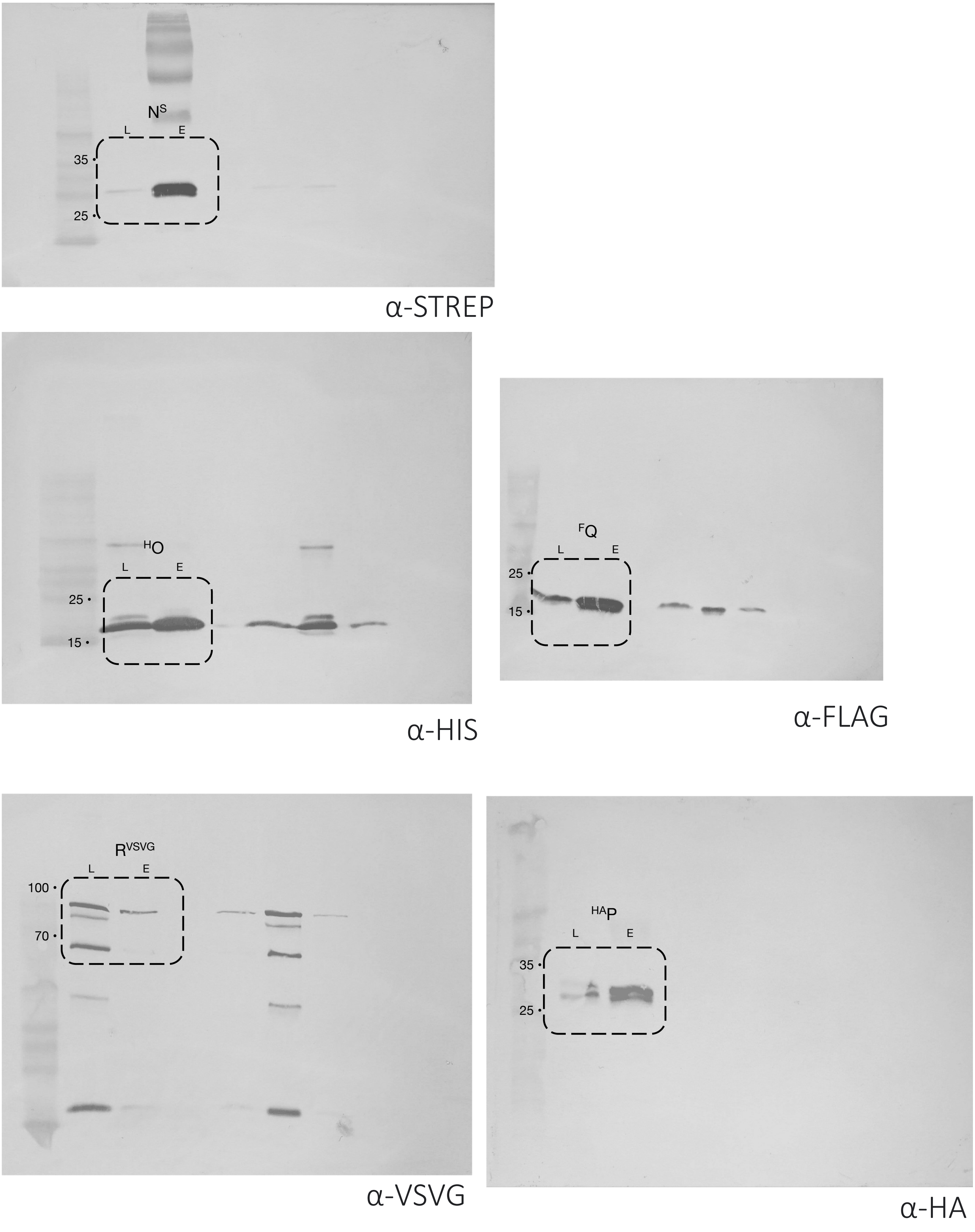

N^S
L
E
35
25
α-STREP
^H O
L
E
25
15
α-HIS
^F Q
L
E
25
15
α-FLAG
R^VSVG
L
E
100
70
α-VSVG
^HA P
L
E
35
25
α-HA

Figure 2d

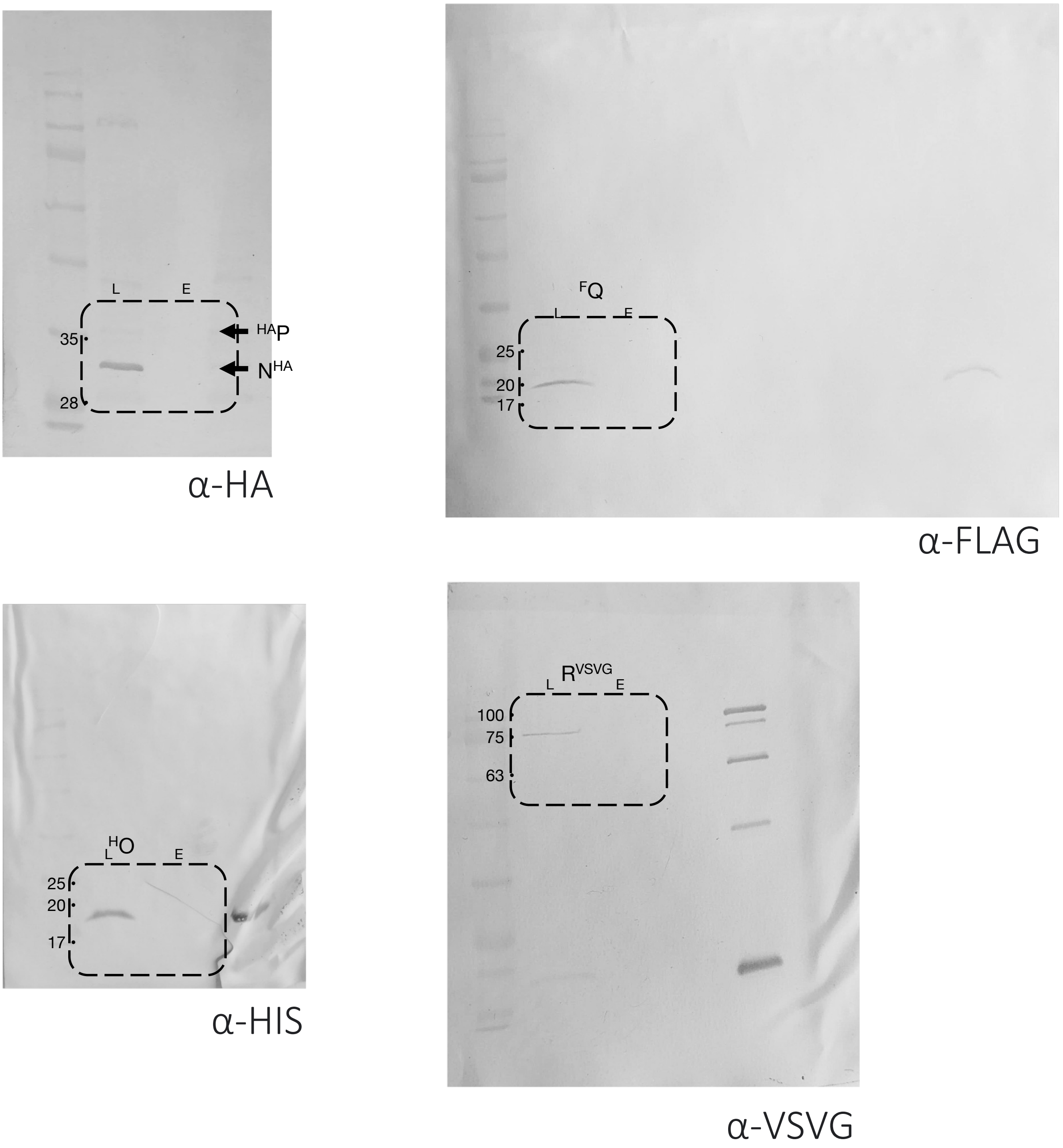

L
E
35
28
HAP
NHA
α-HA
FQ
L
E
25
20
17
α-FLAG
HO
L
E
25
20
17
α-HIS
L
RVSVG
E
100
75
63
α-VSVG

Figure 2d

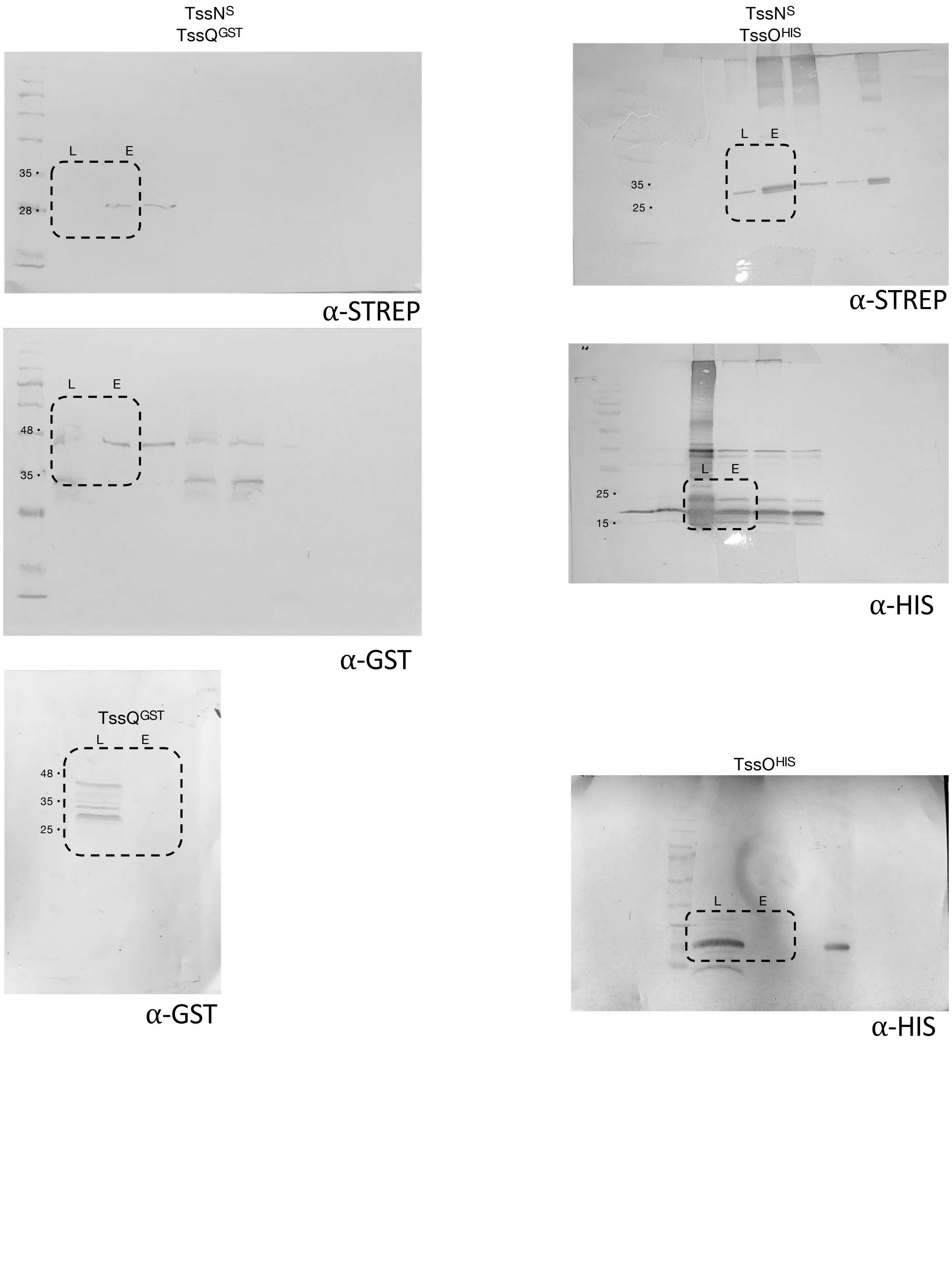
TssN^S
TssQ^GST
L
E
35
28
α-STREP
L
E
48
35
α-GST
TssQ^GST
L
E
48
35
25
α-GST
TssN^S
TssO^HIS
L
E
35
25
α-STREP
L
E
25
15
α-HIS
TssO^HIS
L
E
α-HIS

Figure 3

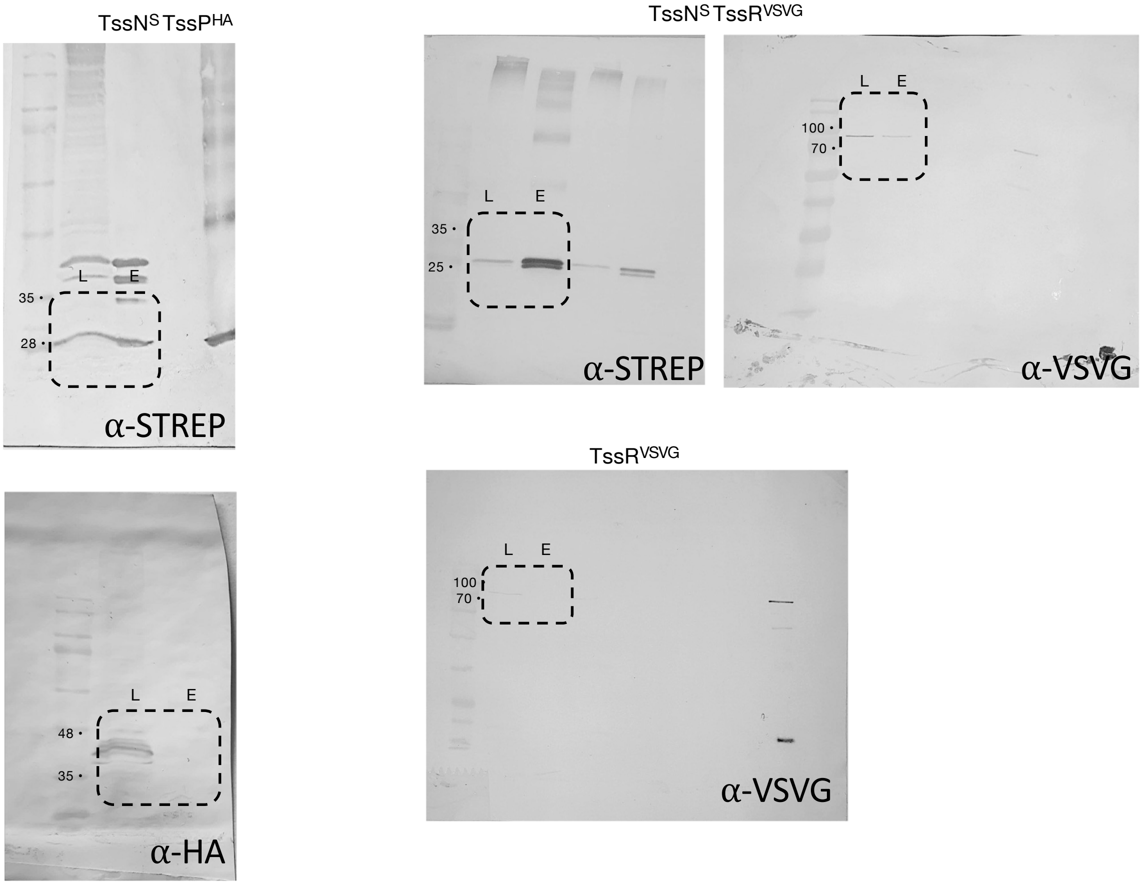
TssN^S TssP^HA
L
E
35
28
α-STREP
L
E
48
35
α-HA
TssN^S TssR^VSVG
L
E
35
25
α-STREP
L
E
100
70
α-VSVG
TssR^VSVG
L
E
100
70
α-VSVG

Figure 3

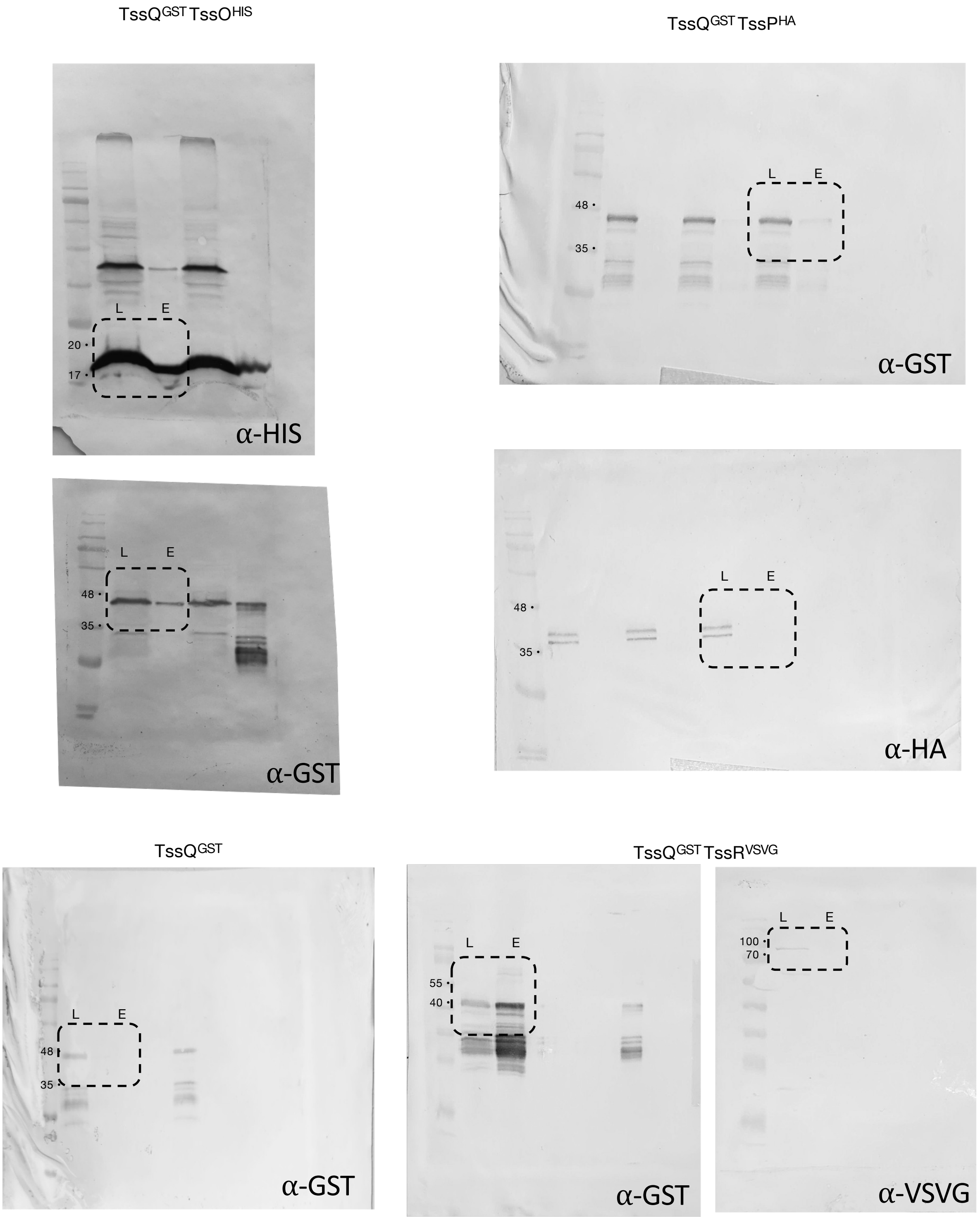
TssQ^{GST} TssO^{HIS}
L
E
20
17
α-HIS
L
E
48
35
α-GST
TssQ^{GST} TssP^{HA}
L
E
48
35
α-GST
L
E
48
35
α-HA
TssQ^{GST}
L
E
48
35
α-GST
TssQ^{GST} TssR^{VSVG}
L
E
55
40
α-GST
L
E
100
70
α-VSVG

Figure 3

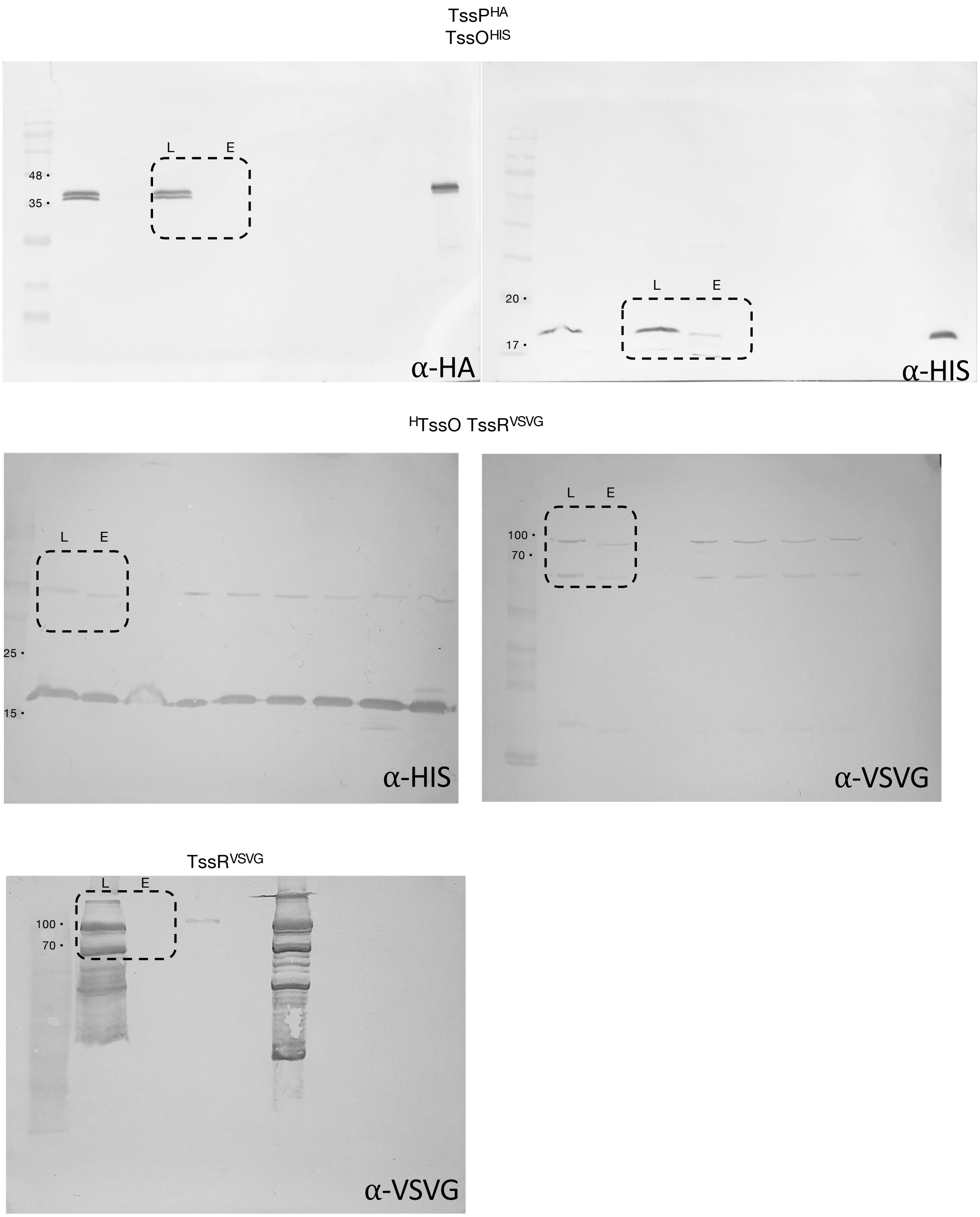

$TssP^{HA}$
$TssO^{HIS}$
L
E
48
35
α-HA
L
E
20
17
α-HIS
$^{H}TssO$ $TssR^{VSVG}$
L
E
25
15
α-HIS
L
E
100
70
α-VSVG
$TssR^{VSVG}$
L
E
100
70
α-VSVG

Figure 3

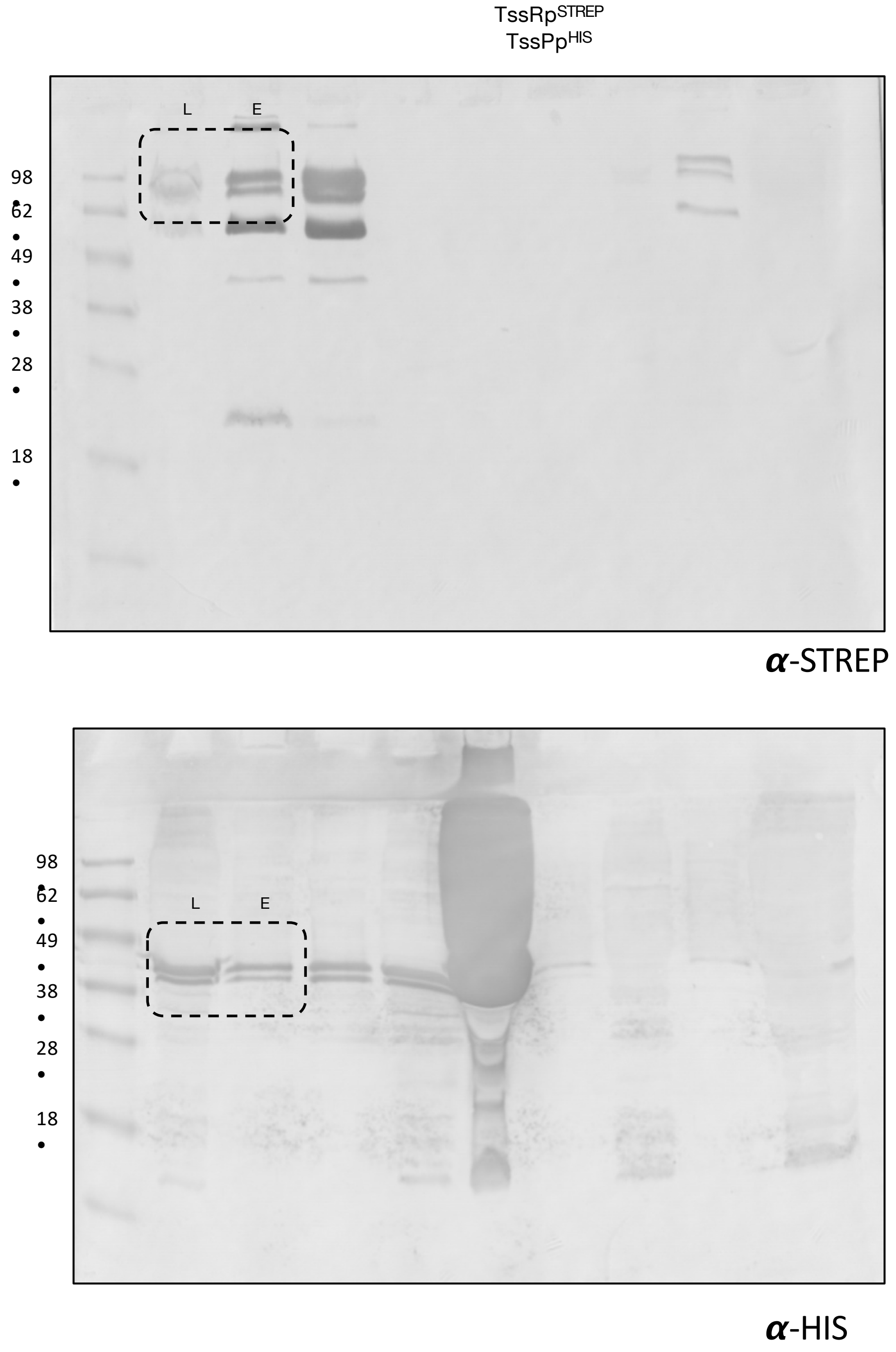
TssRp^STREP
TssPp^HIS
L
E
98
62
49
38
28
18
α-STREP
98
62
49
38
28
18
L
E
α-HIS

Figure 3

TssPp^{HIS}

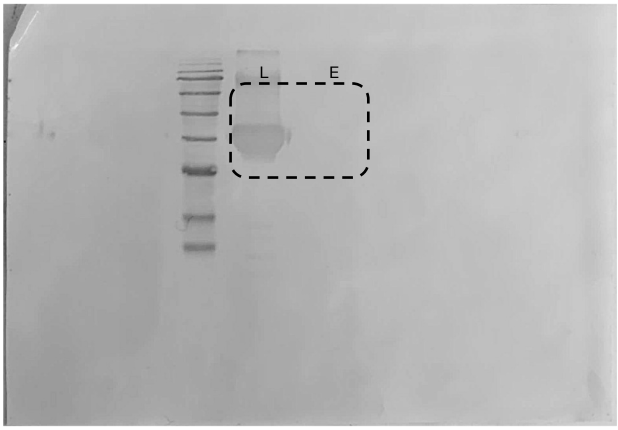

α-FLAG

Figure 3

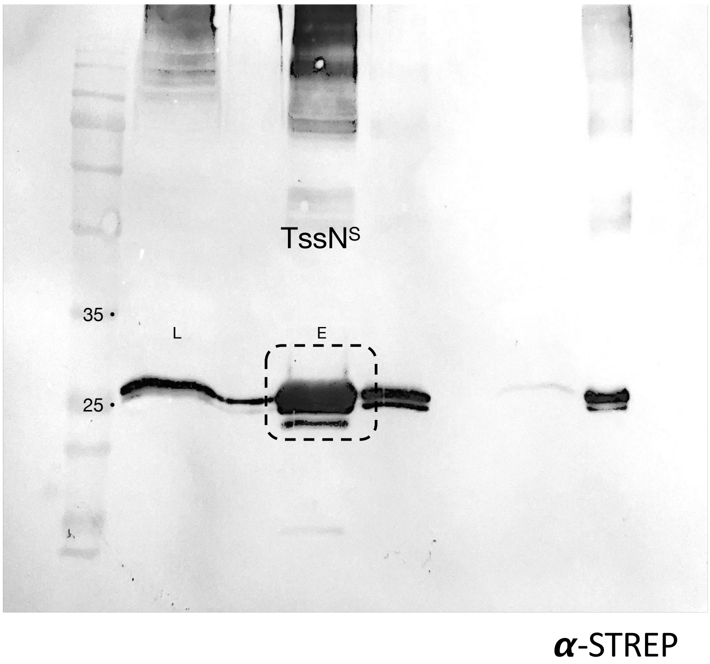

Figure 4a

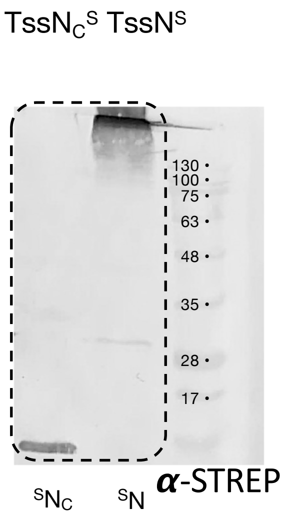

Figure 4d

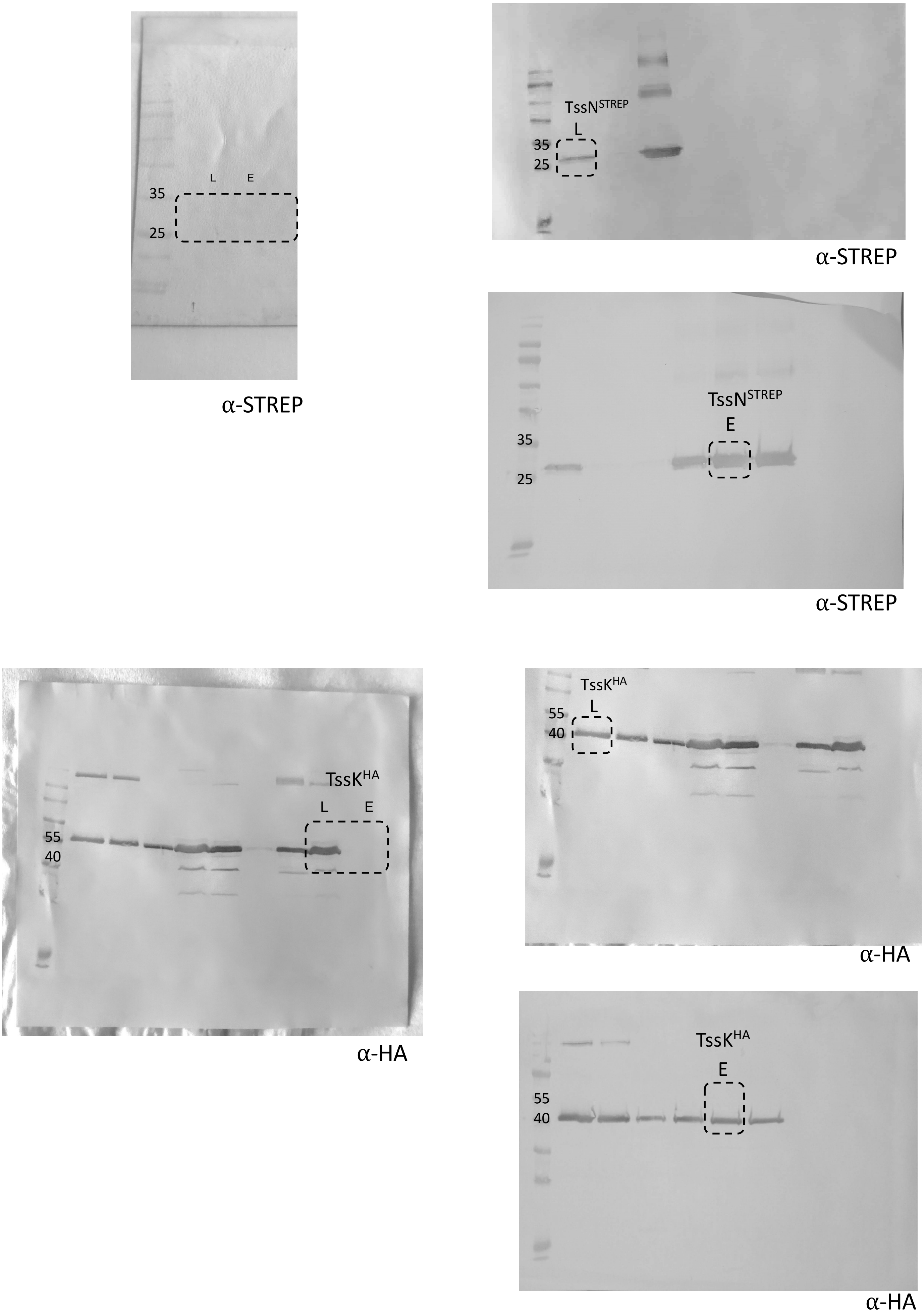
L
E
35
25
α-STREP
TssN^STREP
L
35
25
α-STREP
TssN^STREP
E
35
25
α-STREP
TssK^HA
L
E
55
40
α-HA
TssK^HA
L
55
40
α-HA
TssK^HA
E
55
40
α-HA

Figure 4e

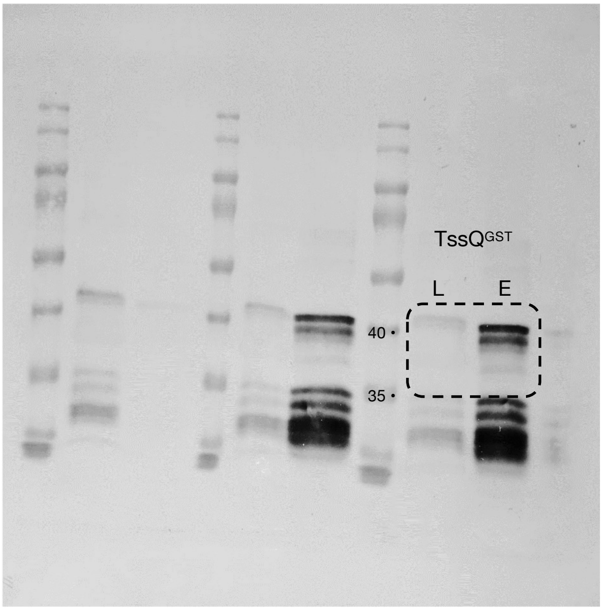

α-GST

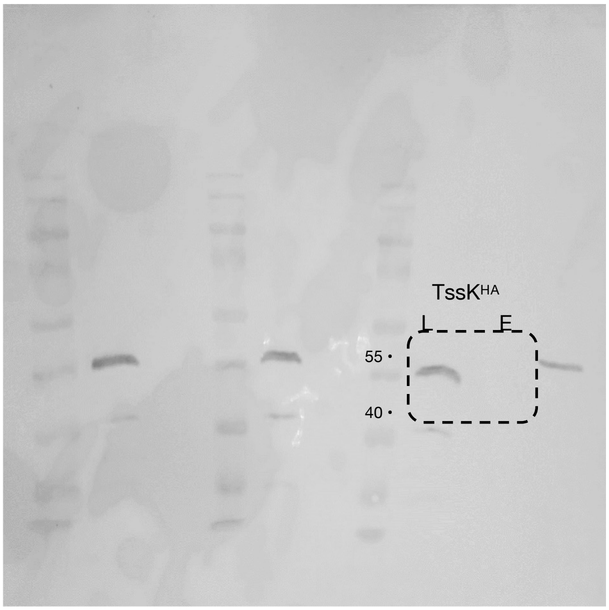

α-HA

Figure 4e
